# LIVING WITH HEART FAILURE AND MULTIMORBIDITY: SEX-BASED DIFFERENCES IN DISEASE BURDEN

**DOI:** 10.1093/geroni/igad104.2968

**Published:** 2023-12-21

**Authors:** Binu Koirala, Xiaoyue Liu, Arum Lim, Chitchanok Benjasirisan, Cheryl Dennison Himmelfarb, Patricia Davidson

**Affiliations:** Johns Hopkins University School of Nursing, Baltimore, Maryland, United States; Johns Hopkins University, Baltimore, Maryland, United States; Johns Hopkins University, Baltimore, Maryland, United States; Johns Hopkins University, Baltimore, Maryland, United States; Johns Hopkins University, Baltimore, Maryland, United States; University of Wollongong, Wollongong, New South Wales, Australia

## Abstract

Heart failure (HF) is one of the leading causes of hospitalization and discharge diagnosis. People with HF present an average of six co-morbid conditions and are mostly older women. Comorbidity or multimorbidity in HF, increases the risk for poor health outcomes, significantly affecting the progression, severity, and complexity of management and care outcomes. This study aimed to examine the sex differences in disease burden among individuals living with multimorbidity. The study is an ongoing cohort study collecting socio-demographic, clinical, and outcome data at baseline, 30 days, and 1-year follow-up. Adults aged 50 years and older living with HF and multimorbidity, defined as the presence of HF and one more chronic condition identified as a common list of comorbidities were recruited using an electronic patient portal. Disease burden data were collected using a modified disease burden impact scale consisting of comorbid conditions included in the eligibility criteria. A preliminary analysis of 406 participants (mean [SD] age = 69.6[9.7] years; 49% female) at baseline found that females (67.7 [9.2] years) were significantly younger than males (71.6 [9.9] years) and females (28.1 [15.2]) had significantly higher disease burden scores compared to males (22.0 [13.6]). An adjusted analysis found females had 3 times higher disease burden scores than males (beta=3.32; 95% confidence interval [CI] = 0.44-6.20, p= 0.024), after controlling for sociodemographic characteristics, social support, resilience, and quality of life. Effective multimorbidity management program requires consideration of both sex and gender-based differences, particularly among older women.

